# Understanding music with cochlear implants

**DOI:** 10.1038/srep32026

**Published:** 2016-08-25

**Authors:** Lisa Bruns, Dirk Mürbe, Anja Hahne

**Affiliations:** 1 Saxonian Cochlear Implant Center, Division of Phoniatrics and Audiology, ENT department, Faculty of Medicine Carl Gustav Carus, Technische Universität Dresden, Dresden D-01304, Germany

## Abstract

Direct stimulation of the auditory nerve via a Cochlear Implant (CI) enables profoundly hearing-impaired people to perceive sounds. Many CI users find language comprehension satisfactory, but music perception is generally considered difficult. However, music contains different dimensions which might be accessible in different ways. We aimed to highlight three main dimensions of music processing in CI users which rely on different processing mechanisms: (1) musical discrimination abilities, (2) access to meaning in music, and (3) subjective music appreciation. All three dimensions were investigated in two CI user groups (post- and prelingually deafened CI users, all implanted as adults) and a matched normal hearing control group. The meaning of music was studied by using event-related potentials (with the N400 component as marker) during a music-word priming task while music appreciation was gathered by a questionnaire. The results reveal a double dissociation between the three dimensions of music processing. Despite impaired discrimination abilities of both CI user groups compared to the control group, appreciation was reduced only in postlingual CI users. While musical meaning processing was restorable in postlingual CI users, as shown by a N400 effect, data of prelingual CI users lack the N400 effect and indicate previous dysfunctional concept building.

Besides language, music is one of the two major acoustic signals for the expression of human nature. Music is a unique property of human kind and is used by all human cultures regardless of training or developmental status. Several features and socio-cultural aspects, like the existence of short phrases in music or group performances, exist as universal aspects of music across cultures^1^,^2^. Listening to music as a normal part of everyday life can bring pleasure and enhance the quality of life^3^.

While music and language both take advantage of the modulation of acoustic parameters to convey information, music is an acoustically more complex stimulus than language, demanding more complex resolution mechanisms^4^,^5^. Music needs the resolution of a wide spectrum of pitch and sound level and contains polyphonic, harmonic and timbral information^6^,^7^. Due to this complexity of musical sounds and the assumption of shared sensory and cognitive mechanisms of language and music processing, music can even function as a cross-modal training stimulus to enhance speech processing^8^.

Music is comprised of multiple different elements. It involves discriminating a tone or a rhythm, and also feeling emotions and liking the sound of a particular piece or instrument. Yet, not a single one of these features can describe the complex entity which forms music as a whole.

For the processing of different musical features, more or less complex mechanisms of perception and information processing are required. Currently, we do not know the exact mechanisms that lead from basic sound perception to the interpretation of sounds as music. Several theories exist on how processing of music can lead to higher and more complex impressions like pleasure or meaning. For example, Zatorre and Salimpoor describe the encoding and storing of tonal patterns as foundational for the complex mechanism of comparison with expectancies and predictions. Furthermore, interactions with subcortical systems while processing music can then result in the impression of pleasure^3^. Music theorists and researchers on music processing describe another complex dimension of music processing, the meaning, i.e., semantic content in music^9^,^10^,^11^,^12^. Up to now, we do not know exactly how meaning is interpreted via music, but several concepts have been proposed^3^,^9^,^13^,^14^. Still, no specific markers (such as rhythm, tempo, harmony) have been identified to transmit a one-to-one musical meaning in a reproducible way. Semantic information is rather dependent on the interpretation by the listener. This bears a resemblance to meta information which is conveyed by “musical aspects” in language (such as timbre of speech, tempo, pauses etc.)^12^. Following some of the main theories on musical semantics, semantic content in music could arise from the interpretation of musical associations either within the music itself (“intra-musical”) or with reference to “musicogenic” and “extra-musical” meaning. Intra-musical meaning would emerge due to expectancies on the music itself which are built up while hearing the musical piece. For example, a sequence of chords would lead to the expectancy of a certain final chord. Musicogenic meaning would arise as a reaction of the human body to music, such as evoked emotions (for instance, sadness felt by the listener) or physical effects (for instance, accelerated heartbeat), while listening to music. Extra-musical meaning would emerge by the interpretation of music in relation to the non-musical world (for instance, a national anthem which serves as a symbol for a country, or musical sounds which resemble a singing bird, or the reference to a state of mind such as happiness)^12^,^14^. Hence, extra-musical meaning is based on an access to preformed meaningful cognitive representations which can be recalled.

Patel *et al.* described that music can convey semantic contents in several ways without referencing a single specific meaning. In contrast to language, there is no basic set of defined meaning in music that we can learn or access at any time, once learned^13^. However, there is a possibility of understanding music cross-culturally and across languages^1^,^2^,^13^. A study from Koelsch *et al.*^14^ investigated if the meaning in music could be perceived similarly across a group of normal hearing (NH) people and identified corresponding electrophysiological markers for extra- and intra-musical meaning^12^,^14^. To test extra-musical meaning processing, they developed music-word pairs and investigated the semantic priming effect of musical pieces on words. Results of this study showed that if the musical piece implied semantic meaning “similar” or congruent to the following word, then the integration process for this word was simplified. First, this study shows that there was meaning in music which was perceived similarly in the majority of participants, and secondly they described the electrophysiological correlates for this process^14^.

In view of the different processing dimensions of sounds and music, it is evident that multiple parameters can influence the handling of information on each other. One could assume that only the correct perception and integration of all the different features of music can lead to the representation we call “music” and which can be pleasurable and full of meaning. With that in mind, it is particularly interesting how people with impaired basic feature perception can get access to music and its information.

Hearing disorders deteriorate the perception of music. Sensory hearing loss occurs on the very basis of acoustical feature perception as a loss of hair cell function. Hair cells usually transform the acoustic mechanical stimulus into electrical signals to stimulate the auditory nerve. Presently, cochlear implants offer a possibility to treat patients with profound or complete sensory hearing loss. A cochlear implant (CI) is a hearing device that enables patients to perceive sounds without using the regular hair cell-transmitted hearing mechanism. An electrode array is inserted in the human cochlea which stimulates the auditory nerve directly by electric impulses. These are produced by an implant which gets preprocessed information through a sound processor that gains acoustic information by a microphone. Currently, CIs have reached a very high technical standard so that understanding speech with the device seems to be achievable for many patients^15^.

Not every patient benefits from the CI in the same way, as the outcome depends (among others) on the hearing experience during childhood. Adults who have had normal hearing experience and hence underwent normal language development prior to hearing loss and cochlear implantation, typically achieve high speech comprehension abilities with CI^15^,^16^. Contrary to these postlingually deafened CI users (henceforth called PostCIUs), there is a group of prelingually deafened adult CIUs (PreCIUs) who were born with profound hearing loss or developed it before or during the period of language acquisition. Receiving the implants in adulthood, PreCIUs rarely develop sufficient auditory language comprehension after cochlear implantation^15^,^16^. The definition of prelingual deafness is not consistent in literature. In many cases, the onset of hearing loss cannot be easily defined, as sensory hearing loss is often more a process than a one-point event. However, articulatory and phonetic deviations in spoken language production are a valuable indication of an early profound bilateral hearing loss^15^.

Obviously, Pre- and PostCIUs have such a different hearing background that they cannot be grouped and compared to a normal hearing control group (NHG). As hearing experience prior to implantation has a major impact on speech comprehension after cochlear implantation, the ability to process and enjoy music may also be influenced^15^. Unfortunately, there is only a handful of studies looking at music perception of PreCIUs who were implanted as adults^17^,^18^,^19^. Even less studies focused on a direct comparison between adult Pre- and PostCIUs^17^,^20^,^21^.

Electrical signal transfer by the CI is generally limited in fine-temporal and fine-spectral as well as dynamic range resolution compared to NH^7^,^22^,^23^. Besides the technical constraints, anatomical changes due to auditory deprivation and hearing experience pre-implantation lead to different individual hearing conditions with the implant^7^,^15^. Bringing this information together, it makes sense that hearing music with a CI is often described as limited and/or not satisfying for CIUs^17^,^24^,^25^,^26^,^27^. However, there are contradictory findings which display that individual CIUs can discriminate in almost the same manner as NH people^28^.

To date, there are two main dimensions of music perception that have been analyzed most in CIUs, discrimination ability and music appreciation^23^,^26^,^29^. Discrimination ability mainly refers to the ability to distinguish isolated stimuli on different dimensions of processing like pure or complex tone discrimination, melody and instrument identification, up to complex stream segregation. It is measured by a variety of psychophysical or electrophysiological approaches^22^,^23^,^24^,^30^,^31^,^32^. Music appreciation depicts a complex process of subjective estimation of the perceived quality of the music. It is usually analyzed using questionnaires or rating systems^17^,^23^,^24^,^25^. However, the complex dimension of meaning in music has not been evaluated in CIUs. Meaning describes a dimension of complex music perception distinct from discrimination or appraisal by evaluation of the perceived semantic content in music^14^. The objective investigation of this dimension is not a binary (“true-false”) condition in regard to meaning in music, making it difficult to measure.

We described earlier that the processing of higher dimensions of music largely relies on learned concepts, expectations and context integration^3^. The process of reaching preformed representations while processing a specific stimulus is reflected in the event-related potential (ERP). Semantic processing of a lot of different stimuli can be studied by investigating a late component in the ERP, namely the N400^33^.

The N400 component represents the difficulty of semantic integration of a stimulus dependent on the previous context. The component is more negative when the perceived stimulus (for instance, a word) is not semantically related to a previously perceived prime stimulus in comparison to a semantically related stimulus. Subtracting the N400 component elicited for related primes from the one elicited for unrelated primes reveals a monophasic negativity around 200–600 ms which is referred to as N400 effect^33^. In the study by Koelsch *et al.*^14^, a N400 paradigm was used to evaluate the processing of musical meaning. They used musical excerpts as priming stimuli followed by visual word presentations. In our study presented here, we used the same music-word pairs as a stimulus set which is described hereafter and in the methods section of the manuscript. The musical primes consisted of complex, mostly classical musical excerpts with an average duration of 10.5 s and a broad variety of pitch and volume. Thus, the semantic integration of complex musical pieces and not of single musical features was evaluated. The musical pieces implied extra-musical meaning references. They either resembled qualities or sounds of objects (for example, a flute playing a songful melody and the corresponding word *bird*), prosodic or gestural cues of the target words (for example, a solemn melody with ascending and descending melody bows and the corresponding word *sigh*) or musical forms that are commonly associated with the target word (for instance, a church hymn and the corresponding word *devotion*). In the Koelsch study, the semantic content of the musical primes was shown to be independent from direct references to language properties (such as the name or the composer of the piece) because the musical stimuli were unknown to the participants. Results in the Koelsch study showed a similar evaluation of the meaning of the musical pieces throughout a majority of the participants (about 80% similar ratings in a behavioral rating), though there was still variability, because musical meaning cannot be unambiguously defined. The processing of this meaning was represented in the N400 component of the ERP which was more negative for incongruent music-word pairs (no music-word priming effect) than for congruent music-word pairs (existent music-word priming effect)^14^. Later studies replicated these results and it was shown that elicitation of semantic associations by music is possible even with very short musical primes of one or two seconds in length^34^,^35^,^36^,^37^. This indicates that the relationship between music and semantic associations is rather robust and may therefore be suitable for studies with CIUs.

With CIUs, ERP studies have been used in several musical contexts, but they mostly looked at the processing dimension of discrimination^38^,^39^. To date, we do not know if the ability of CIUs to perceive meaning in music is restored and if it is represented by the same electrophysiological marker as in a NHG. Semantic meaning, as represented by the N400, has been investigated in CIUs only on the basis of language processing. Those studies showed a comparable N400 effect for semantic language integration processes for PostCIUs as for the NHG^40^. PostCIUs show N400 effects in a language paradigm, and often develop auditory text comprehension with the implant and seem to restore some musical abilities after implantation. Therefore we hypothesized that this group should be able to activate semantic concepts in music. However, for the PreCIUs, early sensory deficits and neural reorganisation processes might limit the possibility of learning and restructuring at adult age^41^. The present study, for the first time, examined the processing of musical meaning as represented in the N400 in CIUs. Thus, we added a third dimension of processing evaluation to the existing dimensions of discrimination and appreciation measurements.

What do we know about the different dimensions of music perception in the different groups of CIUs so far? For the discrimination dimension, most investigations revealed limitations of CIUs regarding discrimination of pitch as well as discrimination and identification of melodies and timbre while perception of rhythm seems to stay relatively unimpaired with CI use^5^,^22^,^24^,^26^,^30^,^31^,^42^. Polyphonic stimuli and complex tones, which need fine spectral resolution, are especially difficult to resolve with CIs^4^. These conclusions were also confirmed by ERP studies for PostCIUs^32^. For the dimension of music appreciation, there is wide variation between CIUs not enjoying music at all and CIUs with increased music appreciation after cochlear implantation^23^,^26^,^43^. PostCIUs typically report dissatisfying music experience post-implantation^25^. Although PreCIUs tend to enjoy music and to rate the quality of music positively, there are limited studies looking at the subjective rating^17^,^18^,^19^,^21^.

We highlight the importance of two main factors when investigating music perception in CIUs: the dimension on which music processing is measured, and the history of hearing loss of the CI population in which music perception is investigated. The aim of this study was to integrate the information about music processing in CIUs in a comparable way. This was achieved by using three methods of measuring different dimensions of music processing and by investigating two different CIU groups. [1] We compared three different dimensions of music processing: discrimination abilities, musical meaning processing and music appreciation. To that end, we conducted a musical discrimination test^30^, an electrophysiological ERP experiment regarding the processing of musical meaning using existing stimuli^14^, and questionnaires assessing music appreciation. [2] We compared two main groups of adult CIUs with a different history of hearing loss (Pre- vs. PostCIUs) with a NHG.

## Results

### Behavioral data: discrimination

To assess the musical discrimination abilities of the participants, we used the MuSIC-test battery. The test was performed by 15 PreCIUs, 38 PostCIUs and 52 NH participants. All groups performed well above chance in all of the subtests except for chord discrimination, where PreCIUs performed below chance level (see Table 1). Data for chord discrimination was normally distributed in all groups as visible in Q-Q plots and verified by Shapiro-Wilk (SW) tests (see Table 2). Pitch, rhythm and melody discrimination as well as instrument identification did not show normal distribution in all of the groups as visible in Q-Q plots and SW tests (see Table 2).

#### Pitch discrimination

**Figure 1:**
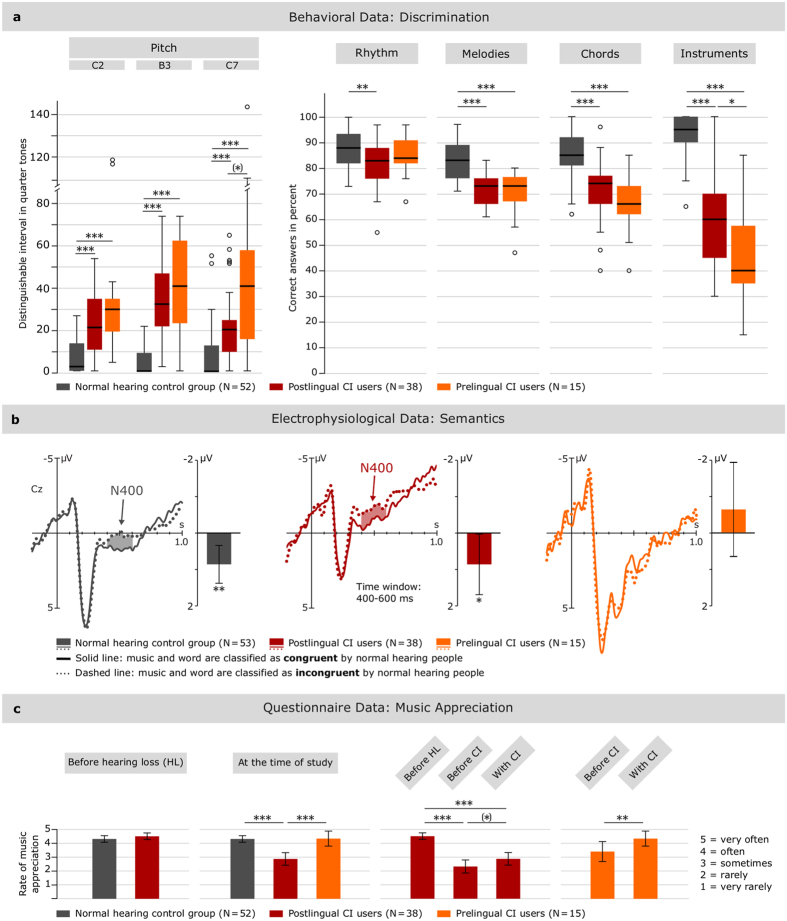
Three dimensions of music processing with CI indicate a double dissociation dependent on hearing experience in childhood. Data is displayed for postlingual CI users (PostCIUs), prelingual CI users (PreCIUs) and the normal hearing control group (NHG). (**a**) Shows behavioral data from a musical discrimination test. (**b**) Shows electrophysiological data of the processing of semantic meaning in music. (**c)** Shows subjective questionnaire data indicating music appreciation. Plots show averaged data across groups. Significant effects in the box and bar plots are marked with asterisks ((*)P < 0.1, *P < 0.05, **P < 0.01, ***P < 0.001). The error bars indicate a confidence interval of 95%. (**a**) On all subtests of the musical discrimination test (excluding rhythm discrimination), both Post- and PreCIUs show significantly lower results than the NHG. Rhythm discrimination is only impaired in PostCIUs. In all subtests (excluding instrument identification), Pre- and PostCIUs do not differ significantly. (**b**) Data shows event-related potentials for the processing of musical semantics on a representative electrode (Cz). Musical pieces served as primes for the processing of visually presented target words which could be semantically related (congruent) or unrelated (incongruent) to the musical pieces. Significant differences between the conditions are marked with shaded areas in the waveforms. PostCIUs display a N400 effect similar to the NHG, indicating that the processing of musical semantic content can be restored in CIUs with previous hearing experience. By contrast, PreCIUs did not show a N400 effect. (**c**) PostCIUs rated their music appreciation significantly lower than both, the NHG and the PreCIU group. Prior to hearing loss, they showed appreciation rates equivalent to the NHG with a significant decrease prior to implantation. PreCIUs showed a significant increase of music appreciation rate following the CI supply resulting in music appreciation rates comparable to the NHG.

Pitch discrimination was conducted for lower (C2), middle (B3) and higher (C7) pitch. In all pitch tests, PreCIUs discriminated the poorest, showing on average the largest discrimination interval and the largest variation, followed by PostCIUs (see Table 3 and left diagram in Fig. 1a). The NHG showed significantly smaller discrimination intervals and ranges (see Table 3). For all pitch tests, statistical analysis revealed significant differences between the groups (Kruskal-Wallis-test [KW], see Table 4). Pre- and PostCIUs differed significantly from the NHG in every pitch test (Mann-Whitney U tests [MWU], see Table 4). Pre- and PostCIUs differed only at C7 with marginal significance (MWU, see Table 4).

#### Rhythm discrimination

PostCIUs rated on average 83 ± 9 (s.d.) % of the rhythm stimuli correctly, while PreCIUs (85 ± 8%) and the NHG (88 ± 6%) reached slightly higher levels (see right diagram in Fig. 1a). Statistical analysis showed that differences between the groups were significant (KW, see Table 4). Further evaluation showed that PostCIUs scored significantly lower than the NHG while differences between PreCIUs and the NHG or between Pre- and PostCIUs were not significant (MWU, see Table 4).

#### Melody discrimination

In the melody discrimination task, PreCIUs rated on average 70 ± 9 (s.d.) % of the melodies correctly, followed by PostCIUs (72 ± 6%) and the NHG (83 ± 7%) (see right diagram in Fig. 1a). Statistical analysis revealed significant differences between the groups (KW, see Table 4). Pre- as well as PostCIUs scored significantly lower than the NHG whereas no significant differences were found between Pre- and PostCIUs (MWU, see Table 4).

#### Chord discrimination

During chord discrimination, PreCIUs on average discriminated the poorest (65 ± 11 [s.d.] % correct) in comparison to PostCIUs (72 ± 13%) and the NHG (85 ± 8%) (see right diagram in Fig. 1a). Statistical analysis showed that group differences were significant (ANOVA, F_(2,102)_ = 27.56, P < 0.0001, η^2^ = 0.35). For the subsequent post-hoc tests Scheffé correction was applied due to homoscedasticity between the groups (F_(2, 102)_ = 2.66, P = 0.08). Pre- and PostCIUs’ results were significantly lower than the NHG’s results (post-hoc tests, PreCIUs/NHG: mean difference = −19.56, P < 0.0001; PostCIUs/NHG: mean difference = −12.64, P < 0.0001). No significant differences were found between Pre- and PostCIUs (mean difference = −6.92, P = 0.10).

#### Instrument identification

In the instrument identification task, PreCIUs rated on average 46 ± 19 (s.d.) % of the instruments correctly, PostCIUs 58 ± 17% and the NHG 94 ± 8% (see right diagram in Fig. 1a). Statistical analysis revealed significant group differences (KW, see Table 4). Pre- and PostCIUs scored significantly lower than the NHG. PreCIUs’ results were also significantly lower than PostCIUs’ (MWU, see Table 4).

### Electrophysiological data: meaning

The ERP experiment was performed by 15 PreCIUs, 38 PostCIUs and 53 NH participants. In this experiment, participants judged whether the meaning of a visually presented word was related or unrelated to the meaning of a previously heard musical piece. The grand average ERP waveforms of all electrode positions are shown in Supplementary Figs S1–3. Analyses of the ERP data included the variables group (PreCIUs/PostCIUs/NHG), condition (congruent/incongruent) and electrode (Fz, Cz, Pz) (see Methods).

#### Behavioral error rates

The error rates in the behavioral judgment task were 38 ± 7 (s.d.) % for PreCIUs, 37 ± 7% for PostCIUs and 19 ± 6% for the NHG. PostCIUs and the NHG performed well above chance while PreCIU's performance was not significantly above chance level (see Table 1, right column). Error rates of all groups were normally distributed as shown by one-sample KS tests (PreCIUs: P = 0.98; PostCIUs: P = 0.97; NHG: P = 0.45). There were significant differences between the groups (ANOVA, main effect group [F_(2,103)_ = 106.50, P < 0.0001, η^2^ = 0.67]). In the following post-hoc tests, Scheffé correction was applied due to homoscedasticity (as shown by Levene’s test: F_(2,103)_ = 1.23, P = 0.30). They revealed that Pre- and PostCIUs both showed significantly higher error rates than the NHG (PreCIUs/NHG: mean difference = 18.94, P < 0.0001, PostCIUs/NHG: mean difference = 17.36, P < 0.0001) while Pre- and PostCIUs did not differ significantly in their error rates (mean difference = 1.59, P = 0.71). Words presented in the incongruent condition showed significantly higher error rates than those presented in the congruent condition (ANOVA, main effect condition [F_(1,103)_ = 17.32, P < 0.0001, η^2^ = 0.14]). No interaction was found between group and condition.

#### ERP data

Descriptively, in PostCIUs and in the NHG, the N400 of the target words showed a more negative excursion when presented in an incongruent musical context than in a congruent musical context (=N400 effect). In PreCIUs the N400 effect was not present (see Fig. 1b). There were significant overall group differences and significant differences in the N400 effect depending on group (see Table 5). The N400 effect was significant for PostCIUs and the NHG, but not for PreCIUs (see Table 6).

#### Properties of the N400 effect

The grand average ERP waveforms of all channels for all groups are shown in Supplementary Figs S1–3. No significant differences were found between the groups regarding the overall scalp distribution of the ERPs. No statistical difference was found between PostCIUs and the NHG in either onset or duration of the N400 effect. As the two CIU groups differed in age, we analyzed the matched NHG including the variable age and did not find any significant interactions involving age and condition. There was no significant correlation between results of the semantic judgement task and the N400 effect (PreCIUs: ρ = 0.16, P = 0.57; PostCIUs: ρ = −0.2, P = 0.10; NHG: ρ = −0.15, P = 0.30). An additional analysis splitting all items in “easy” or “difficult” items, based on the behavioral responses, did not reveal any tendency for a N400 effect for any type of item in the PreCIUs (easy: F < 1; difficult: F_(1,14)_ = 1.40, p = 0.26).

### Questionnaire data: music appreciation

Data was obtained by 15 PreCIUs, 38 PostCIUs and 52 NH participants. The rate of music appreciation after cochlear implantation is shown in Fig. 1c. Data was not normally distributed as visible in Q-Q plots and verified by SW tests (see Table 2). Music appreciation differed significantly between the groups (KW, H_(2)_ = 28.80, P < 0.0001). The average music appreciation rating at the time of study was significantly lower for PostCIUs than for PreCIUs (MWU, U = 116.50, P = 0.001) and the NHG (MWU, U = 387.50, P < 0.0001). PreCIUs and the NHG did not differ in their rating (MWU, U = 365.50, P = 0.69). Before hearing loss, PostCIUs showed comparable music appreciation ratings to the NHG (MWU, U = 860.00, P = 0.24). There was a significant decrease in the rating of music appreciation in PostCIUs comparing the period of NH to the period of hearing loss before cochlear implantation (Wilcoxon signed-rank test [WSR], z = −4.39, P < 0.0001). In their rating after cochlear implantation, they showed only marginal improvement (WSR, z = −1.73, P = 0.08). PreCIUs reported an even higher music appreciation rate after cochlear implantation compared to the time before cochlear implantation (WSR, z = −2.41, P = 0.02).

## Discussion

In this study we looked at three different dimensions of music processing in participants using CIs. While comprehensive analyses about music discrimination abilities and music appreciation in PostCIUs already exist^7^,^23^,^26^,^44^,^45^, data for PreCIUs who were implanted as adults is relatively sparse or non-existent (especially with regard to discrimination abilities or electric brain responses)^17^,^18^,^19^,^20^. Studies comparing these two CIU groups with different history of hearing impairment are extremely rare and have so far only considered music appreciation and music listening habits^17^,^21^. For the first time, we directly compared CIUs implanted as adults with their hearing impairment occurring either pre- or postlingually in regard to three dimensions of music processing: discrimination abilities, processing of musical meaning and music appreciation. Another novelty of the current study was the examination of semantic concepts in CIUs elicited by complex musical pieces being measurable by event-related potentials, i.e., an objective, multidimensional online assessment tool.

In the dimension of discrimination abilities, we observed limitations in CIUs compared to NHG whereas the differences between the two CIU groups were rather marginal (keep in mind the differing group size which might have influenced statistical analysis to some extent). Slight shortcomings were seen in the PostCIUs even for rhythmical processing which is often described as unimpaired^42^. But there was also a large variability with some individuals achieving rather high scores in the rhythm test.

An unexpected finding was that performance of the PreCIUs in the discrimination test was rather similar to the PostCIUs. To date, the former group of CIUs has rarely been studied with regard to music perception. There is general agreement, however, that language comprehension with CI differs largely between Pre- and PostCIUs. PreCIUs did not have a regular oral language acquisition due to their severe hearing loss during childhood. Maturation of auditory pathways are largely reduced with neural reorganization processes being highly probable^46^. These processes, as a consequence of auditory deprivation, have been made responsible for the rather poor language comprehension abilities with CI. Furthermore, the present discrimination performances are better than expected and demonstrate that simple physical characteristics of music are discriminable even for PreCIUs. Only for the identification of instruments, PreCIUs scored significantly lower than PostCIUs which can be explained by the lack of music experience in general and of listening to classical music. Taken together, the data on music discrimination show – in contrast to speech comprehension data – that the onset of hearing loss (pre- vs. postlingual) has a rather marginal impact on discrimination abilities in CIUs.

The core of the present study was the electrophysiological investigation of semantic concepts being activated by complex musical pieces. The electrophysiological approach can broaden our knowledge of music processing in CIUs as it is an objective method with a high temporal resolution uninfluenced by additional behavioral responses. So far, ERP studies on music processing in CIUs have mainly focused on discrimination processes^32^. The current approach extends this spectrum by addressing a higher dimension of musical processing, namely the processing of musical semantics in entire musical pieces with complex sound properties.

In order to elicit a N400 effect in the ERP, i.e., a more negative potential for unrelated music-word combinations compared to related combinations, participants had to access stored semantic concepts. The N400 is generally known as a very stable and well known event-related brain potential related to meaning processing. It can be elicited by an impressively broad variety of linguistic and non-linguistic meaningful stimuli such as, for example, written and spoken words or sentences, odors, pictures, faces, and sounds. The N400 depicts the brain reaction to these kinds of meaningful stimuli, showing an integration process of the stimulus. The amplitude of this component is sensitive to context manipulation. Its character depends on the expectedness of the stimulus, which can be primed by the context (for instance, in this study, musical pieces served as primes for following words). The N400 shows smaller amplitudes for stimuli which were highly expected due to the context, while it shows larger amplitudes when presented in a highly incongruent context. For example, if the word “bird” is spoken and the picture of a bird is shown simultaneously, the N400 amplitude will be smaller than if the picture of a chair was shown simultaneously to the spoken word “bird”. The amplitude therefore shows the effort of the integration process for the presented stimulus^33^.

The N400 has been tested in a broad variety of paradigms with all kinds of meaningful stimuli. In the context of music processing it has been used, for instance, in a NH population to test processing of extra-musical meaning^14^. With CI users we wanted to investigate the dimension of musical meaning not only in a subjective way (with behavioral answers) but our aim was to use the event-related potentials as an objective parameter for musical meaning processing. We did not know if CI users would rate the musical stimuli in a way comparable to the NHG and if the integration process would be represented in the N400 like in the NHG. As hearing with an implant deteriorates the input delivered to the brain for further processing, it was unclear whether semantic information transferred by music is still accessible after cochlear implantation. Data on N400 as a marker for meaning processing in CIUs so far had been narrowed down to language processing^40^. So far, with regard to music processing, only intra-musical but not extra-musical meaning had been tested in CIUs in previous studies^47^ (which is not known to be represented in the N400 parameter).

Studies with NH participants have demonstrated that music can prime meaningful concepts^14^,^34^,^35^,^37^. To be able to make applicable comparisons, we therefore drew on the mentioned stimulus set by Koelsch *et al.* which had already been validated in a NH group. Thereby, it became possible to reveal similarities or differences concerning the electric brain response between the CIUs and the NHG. In the future, it will be important (not only in regard to the higher error rates for CI users) to distinguish between stimuli that are also behaviorally rated similar or different by CIUs and the NHG.

The present data shows that PostCIUs are indeed able to access preformed meaningful representations, despite reduced input information as shown by limited discrimination performance. PostCIUs activate similar associations when listening to music as normal hearing listeners do. Yet, they do not follow their first impression for the later behavioral judgment as the error rate for this group is 37%, which is higher than for the NHG but nevertheless significantly above chance. Thus, they overtly seem to give responses not based on their on-line associations.

By contrast, the PreCIUs do not show an N400 effect. Why do PreCIUs not show this electrophysiological effect despite comparable discrimination abilities? It seems that with regard to semantic concept activation the hearing background pre-implantation, rather than discrimination abilities, makes a significant difference. The PreCIUs investigated in our study showed profound hearing impairment before or during the period of language acquisition. They were implanted after adolescence and therefore had suffered a long period of hearing deprivation with deteriorated acoustic perception. Thus, the semantic concepts for hearing related topics like music possibly could not be formed in the same way as in PostCIUs and the NHG. This is why we assume that the development of hearing related musical semantic concepts had been adversely affected in PreCIUs. Our estimation is supported by a recent study showing the adverse affection of complex relational concepts in adult PreCIUs regarding visual concept formation skills. This study included a cohort of CIUs with prelingual hearing loss and cochlear implantation prior to the age of seven. Hearing status significantly predicted concept formation which was mediated by delayed language and inhibition-concentration skills^48^, i.e., the ability to suppress automatic responses in favor of executing a given non-automatic task.

Further insights on the relationship between deprivation and concept formation come from neurophysiological studies. Auditory brain development needs a combination of nature and nurture for optimal functioning. *In vivo* studies of deaf cats, which received a human cochlear implant, impressively demonstrated central maturation and cortical reorganization processes. In addition, these processes had very circumscribed sensitive periods with several underlying mechanisms^49^. Whereas the auditory pathway up to the auditory cortex is predetermined and capable of some rudimentary feature extraction in deaf animals such as cochleotopy and binaural characteristics, the feature extraction abilities are largely reduced in these animals^41^,^50^. However, feature extraction is one of the main functions of the sensory system and a necessary prerequisite for building object representations. Thus, auditory learning mechanisms are impaired in the congenitally deaf from the very beginning of life^51^. Furthermore, research has shown that especially cortical interactions are much more experience dependent than the afferent auditory system^52^. The development of functional synapses in the cortex and feedback projections is dramatically reduced in deaf animals^41^,^53^.

The identification of auditory objects (e.g., a dog’s bark) needs more than feature extraction. Rather, complex auditory representations have to be built up. These are established via interactions of bottom-up and top-down processes and are modulated by attention^54^. The projections from higher order areas target infragranular cortical layers V and VI, then project to layers II, III and IV and show modulating effects. Deficient stimulation of the primary auditory cortex leads to less synaptic activity in the infragranular layers – though first present in deaf cats in months 2 and 3 - and presumably disconnects early auditory areas from top-down influences^41^,^52^. Consequently, the possibility of selective learning and the development of higher order representations are largely limited in case of auditory deprivation.

Another interesting question arising from our data concerns concept formation skills in PreCIUs who were supplied with CIs early in life as opposed to our PreCIUs being implanted only as adults. If reduced auditory experience is responsible for the deficits, one might expect early implanted children to have the chance to develop semantic concepts similar to NH peers. Further studies are needed to resolve this issue. We have seen that PostCIUs, when listening only with CI, can access preformed semantic auditory concepts like the NHG. But it remains unclear, if those preformed concepts are maintained via the hearing with CI or via a (in some participants) residual hearing and/or a conventional hearing aid of the non-implanted ear. Further studies will be needed to clear this issue. Furthermore, it would be interesting to see the relevant cues in the musical stimuli enabling the PostCIUs to access the semantic representations despite reduced discrimination abilities, e.g. tonality, rhythm or timbre. A systematic testing of several musical properties would further isolate the difficulties of CIUs in music comprehension and might help inform evidence-based training programs aligned to musical semantics.

Apart from the discrimination dimension and the meaning dimension, we additionally approached a third dimension in the current study, namely music appreciation. Remarkably, this analysis dimension proved to be unrelated to the other dimensions and has to be handled independently. The independence of discrimination abilities and music appreciation scores has been mentioned in previous studies^26^. In our study, PostCIUs achieved lower appreciation scores than PreCIUs or the NHG despite being able to activate semantic concepts in music. As their music appreciation was comparable to the NHG before the onset of hearing difficulties, we conclude that these participants compared the current hearing impressions with the unimpaired situation. By contrast, PreCIUs, who cannot compare the hearing impression after cochlear implantation with an unimpaired hearing impression prior to implantation, gain much more positive impressions from the CI compared to their pre-implant situation. Similarly, their score of music appreciation even increases after cochlear implantation as their frequency spectrum and dynamic range expand.

The present study bridges different dimensions of music processing and two groups of CIUs with different hearing histories. Pre- and PostCIUs both showed impairments in discrimination capabilities. Interestingly, an agreement exists that the main predictor of language comprehension in adult CIUs is whether the profound deafness occurred before or after language acquisition. The present data shows that this variable does hardly have any predictive value with regard to music discrimination abilities. The latter seem to be more dependent on CI technology, rather than previous experience. PostCIUs displayed the same electrophysiological pattern for the processing of musical meaning as NH participants. This implies that despite the degraded hearing impression with CI, cortical plasticity enables PostCIUs to access musical semantic concepts, which were built up during the time of NH. By contrast, the influence of prelingual hearing impairment most likely distorted this initial concept formation. Nevertheless, regarding music appreciation, PreCIUs scored as well as the NHG while PostCIUs showed decreased scores. This reflects the difference of the two CIU groups with PostCIUs being able to compare the hearing with CI to unimpaired hearing and PreCIUs enlarging their hearing impression with CI.

Taken together, our results did not exhibit a simple, one-dimensional picture of understanding music with a cochlear implant. Rather, we demonstrated that the discrimination of musical stimuli, the processing of musical meaning and the appreciation of music are different entities of an overall music impression, influencing each other only marginally.

## Methods

The study was approved by the local ethics committee of the medical faculty of the Technische Universität Dresden in accordance with the declaration of Helsinki. All methods were carried out in accordance with the approved guidelines. Informed written consent was obtained of all participants. All participants were German native speakers, had a normal or corrected-to-normal vision and were capable of verbal communication language skills.

### Participants

The participants consisted of 53 CIUs which suffered from prelingual (n = 15) or postlingual (n = 38) hearing loss and a normal hearing control group (n = 53). In all CIUs, cochlear implantation was conducted in adulthood. See Supplementary Table S4 for a description of CIU participant demographics. Sample size justification, especially for the ERP experiment, is given by studies using the same procedures^14^,^34^,^37^,^47^.

#### Postlingual CI users (PostCIUs)

The group of PostCIUs consisted of 38 postlingually deafened patients who were aged from 31 to 79 years (mean 65 ± 10.15 [s.d.]; 21 female). They suffered from acquired profound hearing loss on the implanted ear, which in all cases developed after language acquisition. The contralateral ear showed moderate or severe hearing impairment.

#### Prelingual CI users (PreCIUs)

The group of PreCIUs included 15 adult prelingually deafened patients, aged from 23 to 70 years (mean 36 ± 13.21 [s.d.], 7 female). Criteria for prelingual deafness were the diagnosis of a congenital bilateral profound hearing loss or an onset of profound hearing impairment during early childhood followed by hearing aid supply within this period. In many cases, the definite onset of profound hearing loss could not be determined with certainty for PreCIUs, because there was no objective information achievable for this early point of time. We could only asses a date when the hearing loss was first diagnosed. Therefore, early childhood was defined as diagnosis of hearing impairment prior to six years of age with the assumption that the hearing impairment had already existed prior to three years of age. To make this assumption, we included further criteria to define the PreCIU group which included impaired language production skills regarding articulation and phonation. These criteria were assessed for every CIU participant in an interdisciplinary conference between ENT physicians, speech pathologists and speech rehabilitation therapists who were involved with the patients during the rehabilitation program. All CIUs took part in the study without rewards.

#### Normal hearing control group (NHG)

The NHG served as a control group and consisted of 53 NH participants, matched to the CIUs regarding sex and age (±3 years). The mean age was 56 ± 16 (s.d.) years. All of them had an age-appropriate normal hearing status as controlled by pure-tone audiometry and reported no history of hearing-related, neurological or psychiatric diseases or psychotropic medication. They received payment for their participation. One of the 53 NH participants did not perform the discrimination test and the questionnaire due to personal reasons.

The CI had been monaurally implanted on the right side for 20 CIUs and on the left side for 27 CIUs. It had been bilaterally implanted for 6 CIUs. Out of the 6 bilaterally implanted CIUs, only 3 used both CIs during the measurements. Three bilaterally implanted CIUs used only one CI during the measurements because they were missing sufficient hearing experience with the other CI which had been implanted only recently (a minimum of five months of hearing experience with the CI prior to the measurements was expected).

The CIUs wore implants of different types and processing strategies (23 MedEL^®^ users, 27 Cochlear^®^ users, 3 Advanced Bionics^®^ users). All CIUs participated in a structured rehabilitation program at the Saxonian Cochlear Implant Center, University Hospital Dresden, Germany, which covers speech therapy and fitting of the speech processor after implantation. The mean interval between first fitting of the speech processor and EEG measurement was 18 months (range: 5–103 months).

No participant had received musical training beyond an amateur level, although some had had musical training (defined as instrument or singing lessons outside school in childhood or at present, n = 10 PostCIUs, 16 subjects of the NHG, 0 PreCIUs). In contrast to the PostCIUs, in the group of PreCIUs no one had music experience (defined as performing any recent or former musical activity or receiving former musical training). The amount of music experience was quantified as follows: *1* no music experience (no recent or former musical activity and no recent or former musical training), *2* limited music experience (recent or former musical activity but less than 3 years of musical training), *3* distinct music experience (recent or former musical activity and at least 3 years of musical training). PreCIUs showed on average the least music experience (1.1 ± 0.4 [s.d.]) followed by PostCIUs (1.8 ± 0.8 [s.d.]) and the NHG (2.2 ± 0.9 [s.d.]). This difference between the groups regarding the amount of music experience was significant (KW, n = 106, H_(2)_ = 18.94, P < 0.0001). Subsequent tests revealed a statistically significant difference regarding music experience between Pre- and PostCIUs (MWU, U = 155.50, P = 0.004) as well as between the NHG and PreCIUs (MWU, U = 138.50, P < 0.0001) and between the NHG and PostCIUs (MWU, U = 748.00, P = 0.03). Furthermore, the ranking of preferred musical styles, which were inquired by questionnaires, differed between the CIU groups. To measure the preferred musical styles, the participants were asked to select which kind of musical styles they listened to from a list of 8 styles (classical music, rock music, pop music, country & folk music, choir music, church music, electrical music and Jazz & Blues). The evaluation of selections showed that PreCIUs did not rank classical music as one of their three preferred styles of music while PostCIUs ranked classical music as their second preferred style.

### Assessment of musical discrimination abilities

#### Stimuli and presentation

To assess the musical discrimination abilities of the participants, we used the MuSIC-test battery^30^. Five categories were evaluated in subsets: pitch, rhythm, melody and chord discrimination as well as instrument identification. For each subtest a representative subset of the existing battery was created and presented completely, but in a randomized order, to every participant.

For pitch discrimination, natural string sound was used for presentation. It had the advantage of a relatively long-lasting tone and a natural sound spectrum. Also, complex pitch seems to be easier to identify when presented by strings than by, for instance, piano^5^. The files had initially been normalized to −3 dB and the length of the notes adjusted to a variation of no more than 10%. Three pitch levels were tested (C2, B3 and C7) in an intertwined way. Two tones were presented in succession with the second tone being either higher or lower than the first. Participants had to judge in a two-alternative forced-choice procedure, whether the second tone was lower or higher than the previous one. The starting interval was set to 18 quartertones for CIUs and 10 quartertones for the NHG. The threshold interval was approached using an adaptive staircase algorithm following the procedure by Levitt (1971)^55^. The confidence level was set to 79%. In this way, for each pitch level, an interval was determined which could be at least discriminated by the participant.

For rhythm discrimination, a representative subset of 34 pairs of rhythms in different categories of difficulty was presented. Timbre was equally distributed between snare drum, bongos, woodblock and timpani. Changes in rhythm for the “different” file could be either in the amount or duration of tones and breaks.

For melody discrimination, a subset of 42 melodies was built. It contained melodies at different levels of difficulty. Four different instruments were used representing different timbre and pitch: piano (12 pieces), cello, violin and transverse flute (each 10 pieces). Changes in melody could be slightly (for instance only one tone changed) to severely (up to every tone changed) difficult while rhythm was constant.

Chord discrimination was tested using piano files presenting chords with different levels of difficulty. Changes in the “different” chord ranged from only one tone to the whole construction or pitch of the chord.

For instrument identification, a subset was built containing four different instrument families with representatives of high and low pitch: strings (violin, cello), woodwinds (traverse flute, bass clarinet), brass (trumpet), keyboard instrument (piano), plugged instrument (guitar), percussion instruments (xylophone, snare drum) and organ. Each instrument played two melodies in total. Once an easy nursery melody was played (“Baa baa black sheep”) and once a typical piece for the respective instrument. The order of the instruments and of the musical pieces was random.

#### Procedure

The test was conducted in a quiet room with a laptop presenting mono-files. For the patients, the sound stimuli were presented unilaterally to the implanted ear via headphones (*HD201, Sennheiser, Wedemark, Germany*) covering the CI microphone as well as the contralateral ear. The latter was additionally masked by an ear plug to deactivate possible residual hearing. For the NHG, the stimuli were presented bilaterally using the same headphones. The reason for this was that monaural stimulation in bilaterally normal hearing adults is unusual and needs getting used to. Rather than introducing the additional “task” to listen with only one ear, which would influence their performance in the main task, we tested them in their usual, bilateral hearing situation. Sound level was adjusted individually for every participant, starting from a standard level of 70 db SPL at 500 Hz, so that a comfortable hearing impression was possible. CIUs were asked to use the same settings of the CI sound processor as for the ERP experiment. Before starting each subtest, it was explained by written and verbal instruction to each participant including examples guaranteeing task understanding. For the instrument identification test, no examples were provided. For pitch discrimination, two tones were played subsequently with a short break in between. After the presentation, the participants had to choose between two buttons showing either two descending or two ascending notes for the first or second tone to be higher. After a button press, the next trial started. The test stopped only after the threshold interval was determined, although breaks were always allowed. For rhythm, melody and chord discrimination, in each trial two stimuli were presented sequentially with a short break in between. Participants were asked to choose whether the two stimuli were the same or different by pressing one out of two buttons. A maximum of two repetitions was allowed. For instrument identification, a solo piece was played, while all of the ten possible instruments were presented as pictures on the screen. After the presentation, the participants had to choose the picture from the closed set which showed the playing instrument. The array of the instrument pictures on the screen changed in a randomized way. Pressing the button started the next trial. The test stopped when all trials were executed. No feedback was given until the termination of the entire test.

#### Data analysis

For each subtest a single value was obtained for every participant (intervals for pitch tests, percentages for the other subtests). Data was analyzed using the software *SPSS (IBM SPSS Statistics Version 23 for Windows, IBM, Armonk, USA).* For every group and every subtest, Q-Q plots were evaluated and Shapiro-Wilk (SW) tests were conducted to test the sample distribution against standard normal distribution. For normally distributed variables, a step-down analysis was applied: the impact of overall group differences was tested by a global analysis of the factor group (PreCIUs/PostCIUs/NHG). In the case of significant differences between groups, further parametric methods, i.e., ANOVAs and post-hoc tests or t-tests, were applied. For not normally distributed data, non-parametric tests were applied in a step-down-analysis. To test global group differences, Kruskal-Wallis one-way analysis of variance (KW) was conducted. This test was used to compare the three independent group samples together in one analysis. If subsequent analyses included only two groups, Mann-Whitney U tests (MWU) were conducted. Note the different group sizes which might have influenced the statistical analysis to some extent.

### Assessment of meaning processing using event-related potentials

#### Stimuli

Stimuli were taken from Koelsch *et al.*^14^. They consisted of 44 musical pieces used as primes and 44 words used as targets. The whole stimulus set can be found on the website www.stefan-koelsch.de, linked to the original paper^14^. The musical pieces and the target words had been designed by Koelsch *et al.* as follows: In a pre-experiment a set of target words and musical pieces had been constructed and participants had rated the semantic fit between the music and two possible target words (one related and one unrelated) on a scale ranging from −5 (semantically strongly unrelated) to +5 (semantically strongly related). Every musical piece therefore had two ratings, one for a related and one for an unrelated target word. Only musical pieces with significantly different ratings for the related and unrelated target word where chosen as stimuli for the following ERP experiment.

The musical pieces were complex pieces of classical music (mean duration 10.5 s) covering a broad variety of pitch, rhythm, instrumentation and dynamic cues. They were downloaded as mp3 files from the website of the author (www.stefan-koelsch.de) and prepared to be used in the presentation software. First, the 95^th^ percentile of the averaged SPL of each sample was measured by an SPL meter. Further, this measure of each sample was adjusted to a standard SPL level which corresponded to the mean SPL level of all non-normalized stimuli using the freeware software *Audacity*^*®*^. Hereafter, the musical stimuli were converted to wave files (16 bit) for presentation purposes. Five additional pieces were taken as example stimuli. The target words were all German nouns. In the EEG experiment, a total of 88 trials were presented, each containing a musical piece as a prime followed by a word as a target. Every musical prime was presented twice, once followed by a meaningfully related target word, once by a meaningfully unrelated target word. The unrelated musical prime-target relations had been constructed by interchanging the targets of the related combinations. Thus, every target word was presented twice, once in a related and once in an unrelated context.

#### Procedure

**Figure 2:**
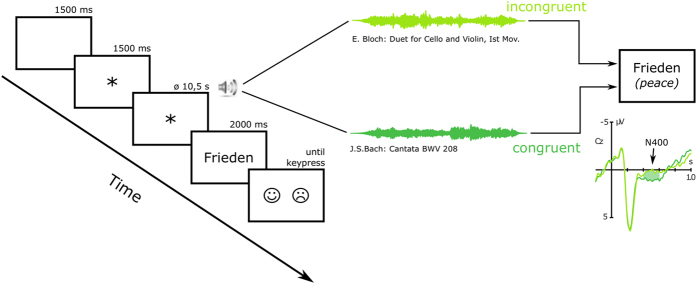
Illustration of design and procedure of the current study. The monitor first showed a blank screen, followed by a fixation mark, the target word and a sign showing a happy and a sad face. The presented musical excerpts of 10.5 seconds on average were either semantically congruent or incongruent to the visually presented target word (based on pre-tests by Koelsch *et al.*^14^). ERPs were measured to the visual words. The same target word was used for the congruent and for the incongruent condition. Participants judged the semantic relatedness of music and word by pressing a key (referring to smiling or sad emoticon). The keypress also started the next trial.

Participants were seated in a comfortable chair in a quiet room, 150 cm in front of a computer screen. Auditory stimuli were presented via loudspeakers with a set sound intensity of 65 dB SPL. CIUs were asked to choose the processor program which they used in everyday life to listen to music and to adjust volume and microphone sensitivity to standard settings. In case patients usually used a conventional hearing aid on the contralateral side, this was switched off during the experiment. Potential residual hearing was muted by ear plugs (*Bilsom 303L*, *Howard Leight*) and soundproofing headphones (*3M Peltor Optime II H520A*, *3M*) with the CI microphone attached to the outside. CIUs were asked to use the same settings of the CI sound processor as for the musical discrimination test. See Fig. 2 for an illustration of the design and procedure of the ERP experiment. Each trial started with a blank screen for 1500 ms, followed by a fixation signal (*) appearing on the screen 1500 ms before onset of the musical stimulation. The fixation signal remained visible until it was replaced by the target word appearing immediately after the end of the musical piece. The target word was visible for 2000 ms until a response cue appeared. Participants were required to judge the stimuli for congruency by pressing one of two buttons during the presentation of the response cue. The response of the participant set off the start of the next trial (cf. Fig. 2).

The trials in the ERP experiment were presented in two presentation blocks, each block including 44 music-word pairs. Trials were presented in a pseudo-randomized order with the following constraints: the minimal inter stimulus interval between two presentations of the same musical piece was set to thirty items; no more than three congruent (resp. incongruent) music-word pairs were presented in a row before an incongruent (resp. congruent) music-word pair was presented; in each of the two presentation blocks just as many congruent as incongruent trials were presented. The randomization process provided three trial lists which were then presented forwards and backwards resulting in six different trial sequences in a pseudo-randomized order. The presentation of those trial sequences was equally distributed throughout the participants.

Prior to the start of the experiment, participants received written instructions and five example trials were presented. Participants were asked to avoid movements and eye movements during the presentation of the target words.

#### Electrophysiological recordings

The EEG was recorded simultaneously with 9 monopolar Ag/AgCl electrodes mounted in an elastic cap (*EasyCap GmbH, Herrsching, Germany*). In accordance with the 10–20 system, Fz, Cz, Pz, F3/4, C3/4, P3/4 as well as left and right mastoid positions with Cz serving as online reference were measured. Adhesive paste was used to guarantee correct placement and electrical connection to the scalp. Bipolar EOG recordings were obtained from electrodes above and below the right eye and from the outer canthi of the left and right eye. Electrode impedance was kept below 5 kOhm. The biosignals were amplified within a bandpass from DC to 40 Hz and digitized with 512 Hz. For CIUs, the data was re-referenced offline to the mastoid electrode contralateral to the implanted side, for the control group to an average of the two mastoid signals.

#### Data analysis

The raw data was analyzed using the software EEProbe EEProbe (*ANT Neuro, Enschede, Netherlands*). ERPs were computed for each participant in both experimental conditions (congruent/incongruent) for 1000 ms segments time-locked to the onset of the target words. Each segment was computed relatively to a pre-stimulus baseline of 200 ms. Trials with typical EOG movement artifacts were corrected using an EOG correction tool in EEProbe. All other trials contaminated by ocular, movement or electrode artifacts were rejected. Note that the ERPs are not likely to be influenced seriously by CI artifacts, as these results refer to the target word which is presented visually in succession to the auditory presentation. Rejections were equally distributed across the three participant groups and the two conditions (main effects and interactions: all F < 1). Trials with incorrect behavioral response were excluded from ERP analysis. The average number of trials included in the average was 30.5 ± 5.2 (s.d.) for PreCIUs, 26.6 ± 3.5 (s.d.) for PostCIUs and 33.9 ± 4.0 (s.d.) for the NHG.

To evaluate N400 effects, statistical analyses of mean area voltages between the waveforms for incongruent and congruent trials for each group and condition were performed in a time window ranging from 400 to 600 ms. Electrodes with central or lateral position were analyzed separately to quantify topographic differences. Generally, Greenhouse-Geisser correction was applied when evaluating effects with more than two degrees of freedom in the numerator. Here, we report uncorrected degrees of freedom and corrected probabilities.

##### Central electrodes

All analyses were quantified using multivariate analysis methods with the between subject variable group (PreCIUs/PostCIUs/NHG) and the within subject variables condition (congruent/incongruent) and electrode (Fz/Cz/Pz). Step-down analyses and post-hoc tests were conducted in case of significant two- or threefold interactions (P < 0.05) including the variables group, condition and electrode.

##### Lateral electrodes

The multivariate analysis contained both condition and group variables (see above for grading) and additionally the variables hemisphere (left [F3, P3]/right [F4, P4]) and region (anterior [F3 & F4]/posterior [P3 & P4]). Relevant interactions included the variables condition and group in interaction with hemisphere and/or region. In the case of relevant two-, three- or fourfold interactions, further step-down analyses and post-hoc tests were conducted.

To evaluate onset and duration of the N400 effect, two further time windows were introduced, reflected in the variable time window. For analyzing the onset of the N400 effect the time windows 320–400 ms and 400–600 ms were used. For analyzing the duration of the N400 effect, the time windows 400–600 ms and 650–750 ms were used.

### Assessment of music appreciation

To assess music appreciation, a questionnaire was designed. Participants were asked to rate the frequency of appreciating music when listening to it, independent of the amount of time they spent listening to music or the kind of music they listened to. The participants chose from a Likert-score ranging from 1 (very seldom) to 5 (very often). For CIUs, three time points were requested: 1. prior to hearing loss (only for PostCIUs), 2. prior to cochlear implantation (i.e., with hearing loss), 3. with CI at the time of study. Due to not normally distributed data, analysis was conducted using nonparametric tests in a step down analysis. KW tests were followed by MWU tests for comparisons between groups. When comparing two time points of one variable in one group, Wilcoxon signed-rank tests (WSR) were applied.

## Additional Information

**How to cite this article**: Bruns, L. *et al.* Understanding music with cochlear implants. *Sci. Rep.* **6**, 32026; doi: 10.1038/srep32026 (2016).

## Supplementary Material

Supplementary Information

## Figures and Tables

**Table 1:** Test against chance level for data of the musical discrimination test and for the behavioral error rate in the ERP experiment (one sample t-test for each group).

Subtest		Rhythm	Melodies	Chords	Instruments		Error rate
*Test level (%)*		*65*	*62*	*67*	*20*		*59*
PreCIUs	***T***_***(14)***_	10.07	3.48	−0.61	5.37	***T***_***(14)***_	1.51
	***P***	<0.0001*	0.004*	0.55	<0.0001*	***P***	0.16
PostCIUs	***T***_***(37)***_	12.16	9.81	2.51	13.65	***T***_***(37)***_	4.05
	***P***	<0.0001*	<0.0001*	0.02*	<0.0001*	***P***	<0.0001*
NHG	***T***_***(51)***_	26.69	21.84	15.22	68.23	***T***_***(52)***_	25.37
	***P***	<0.0001*	<0.0001*	<0.0001*	<0.0001*	***P***	<0.0001*

^*^Group result is significantly above chance level.

**Table 2:** Test of normality for data of the musical discrimination test and for music appreciation data (Shapiro-Wilk test, alpha level of 0.05).

Subtest		Pitch C2	Pitch B3	Pitch C7	Rhythm	Melodies	Chords	Instruments	Music appreciation
PreCIUs	***W***_***(15)***_	0.68	0.92	0.89	0.95	0.88	0.96	0.95	0.73
	***P***	<0.0001	0.20†	0.06†	0.57†	0.04	0.69†	0.55†	<0.001
PostCIUs	***W***_***(38)***_	0.95	0.97	0.89	0.94	0.90	0.97	0.97	0.87
	***P***	0.06†	0.38†	0.001	0.04	0.003	0.46†	0.43†	<0.0001
NHG	***W***_***(52)***_	0.81	0.79	0.67	0.95	0.96	0.96	0.77	0.74
	***P***	<0.0001	<0.0001	<0.0001	0.03	0.06†	0.10†	<0.0001	<0.0001

^†^Data show normal distribution.

**Table 3:** Results of the Pitch discrimination test for three pitch levels (C2, B3, C7) and for the three groups.

Subtest	Pitch C2	Pitch B3	Pitch C7
PreCIUs	**30** (34/5–119)	**41** (25/1–74)	**41** (41/1–144)
PostCIUs	**22** (14/1–54)	**32** (18/3–74)	**21** (17/1–70)
NHG	**3** (8/1–27)	**1** (6/1–22)	**1** (13/1–57)

Median distinguished interval in quartertones (s.d. / range).

**Table 4:** Statistical analysis of group differences of the Musical discrimination test (non-parametric tests).

Subtest		Pitch C2	Pitch B3	Pitch C7	Rhythm	Melodies	Instruments
KW^1^: PreCIUs/PostCIUs/NHG	*H*_(*2*)_	40.48	58.94	27.24	9.82	42.73	72.28
	*P*	<0.0001*	<0.0001*	<0.0001*	0.007*	<0.0001*	<0.0001*
MWU^2^: PreCIUs/NHG	*U*	53.50	77.50	137.00	290.00	97.50	8.50
	*P*	<0.0001*	<0.0001*	<0.0001*	0.13	<0.0001*	<0.0001*
MWU^2^: PostCIUs/NHG	*U*	363.50	115.50	464.50	614.00	267.50	68.00
	*P*	<0.0001*	<0.0001*	<0.0001*	0.002*	<0.0001*	<0.0001*
MWU^2^: PreCIUs/PostCIUs	*U*	214.50	229.50	198.00	251.00	271.00	175.50
	*P*	0.16	0.27	0.09	0.50	0.78	0.03*

^1^Kruskal-Wallis-test. ^2^Mann-Whitney U test.

^2^Mann-Whitney U test.

^*^Significant group differences. Statistics for Chord discrimination data (parametric tests) are shown in the main text.

**Table 5:** Statistical analysis of the ERP data.

Effect	df	F	P	η^2^
Condition (Cond)	1, 103	1.72	0.19	0.02
Electrode (Elec)	2, 206	25.98	<0.0001*	0.20
Group	2, 103	3.06	0.05*	0.06
Cond × Group	2, 103	3.62	0.03*	0.07
Elec × Group	4, 206	2.7	0.05*	0.05
Cond × Elec	2, 206	1.04	0.34	0.01
Cond × Group × Elec		<1		

ANOVA containing the variables group, condition and electrode. Analysis of the ERPs measured on Fz, Cz and Pz electrodes in the time window of 400–600 ms. Greenhouse-Geisser correction of the P-value was used for inner subject effects (condition and electrode).

^*^Significant main effects and interactions.

**Table 6:** Subsequent analysis of the significant interaction between group and condition: condition effect in the three groups.

Group	*df*	*F*	*P*	*η*^*2*^
PreCIUs	1, 14	1.53	0.24	0.10
PostCIUs	1, 37	4.89	0.03*	0.12
NHG	1, 52	10.45	0.002*	0.17

One-way ANOVA containing the variable condition. Analysis of the ERPs measured on Fz, Cz and Pz electrodes in the time window of 400–600 ms. Greenhouse-Geisser correction of the P-value was used.

^*^Significant condition effect.
