# Supplementary material for: Understanding music with cochlear implants
Source: Sci Rep. 2016 Aug 25;6:32026. doi: 10.1038/srep32026 (PMC4997320; doi:10.1038/srep32026)
Supplement: Supplementary Information [file srep32026-s1.pdf]

Supplementary information
Understanding music with cochlear implants

Lisa Bruns, Dirk Mürbe, Anja Hahne*

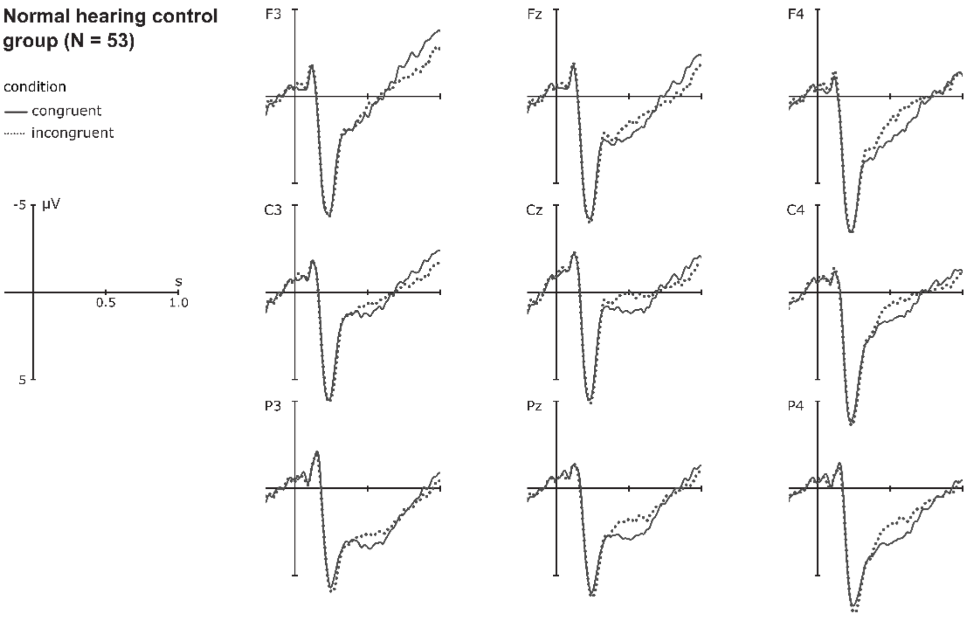

Supplementary Figure S1 Event-related potentials depict the processing of words preceded by either congruent or incongruent musical excerpts. Grand average ERPs are shown for the normal hearing control group on all measured electrode positions. ERPs for incongruent music-word-stimuli show a more negative excursion between 400 – 600 ms than ERPs for congruent stimuli. This N400 effect reflects the processing of musical meaning in the normal hearing control group.

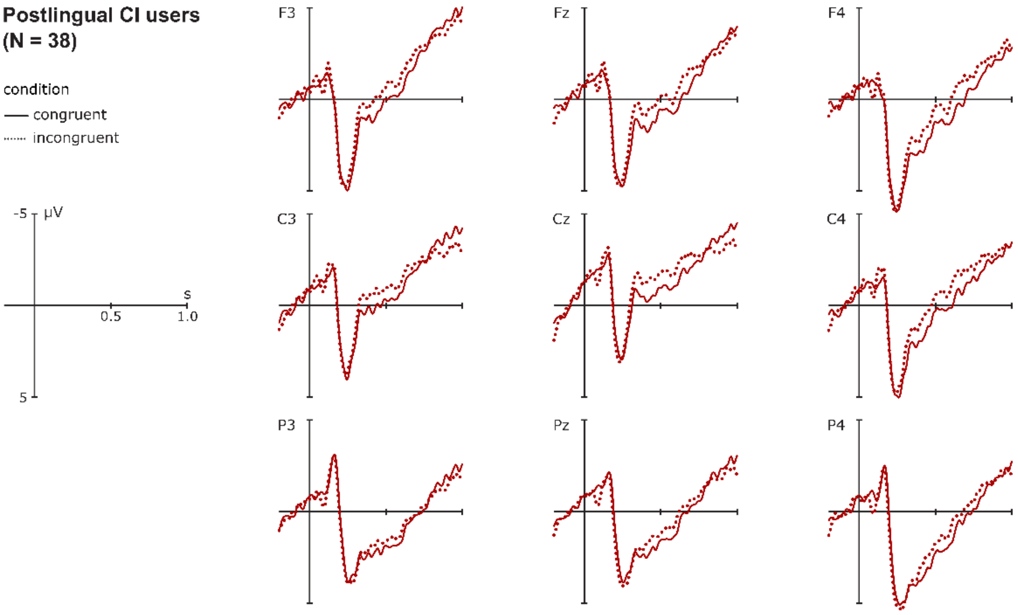

Supplementary Figure S2 Event-related potentials depict the processing of words preceded by either congruent or incongruent musical excerpts. Grand average ERPs are shown for the postlingual CI user group on all measured electrode positions. ERPs for incongruent music-word-stimuli show a more negative excursion between 400 – 600 ms than ERPs for congruent stimuli. This N400 effect reflects the processing of musical meaning and indicates restored access to semantic musical representations with the hearing impression of a CI in postlingual CI users.

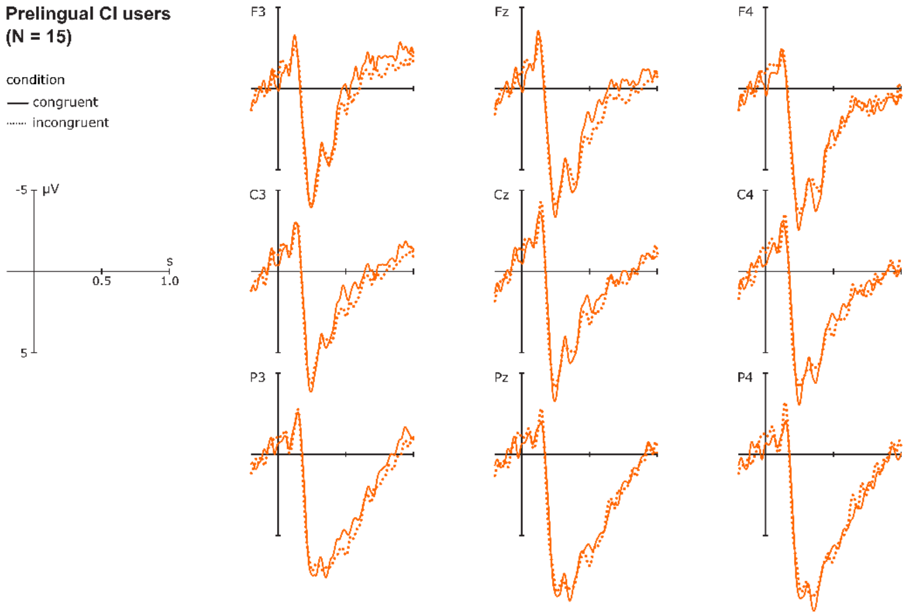

Supplementary Figure S3 Event-related potentials depict the processing of words preceded by either congruent or incongruent musical excerpts. Grand average ERPs are shown for the prelingual CI user group on all measured electrode positions. No difference is found in the ERPs for incongruent and congruent music-word-stimuli between 400-600 ms. The absent N400 effect indicates missing access to semantic musical representations with CI by the prelingual CI user group probably due to dysfunctional concept formation in childhood.

Supplementary Table S4 CIU participant demographics and speech comprehension data measured by *Freiburger Einsilbertest*.

Number	Gender	Age	Group	Aetiology of hearing loss	CI type (implant / processor)	Age at onset of profound hearing loss	Age at implantation	Months of CI experience	Contralateral use of hearing aid	Numbers 65db	Words 65db
1	f	73	postCIU	hereditary	Concerto / Opus 2	55	72	13	-	60	0
2	f	72	postCIU	unknown	Concerto / Opus 2	40	71	10	+	90	30
3	f	68	postCIU	acoustic neuroma	CI422 / CP810	67	67	10	+	80	30
4	f	74	postCIU	drug toxicity	Concerto / Opus 2	73	73	10	+	70	50
5	f	47	postCIU	unknown, poss. hereditary	CI422 / CP810	-	46	5	+	100	90
6	f	71	postCIU	ISSHL	Concerto / Opus 2	68	70	12	-	90	10
7	f	57	postCIU	otosclerosis	CI422 / CP810	52	56	13	+	100	95
8	f	71	postCIU	unknown, familial	Concerto / Opus 2	64	70	11	+	40	5
9	f	66	postCIU	ISSHL	Concerto / Opus 2	61	65	10	+	100	50
10	f	70	postCIU	ISSHL	CI512 / CP810	35	68	21	-	100	70
11	f	31	postCIU	prenatal toxoplas-mosis, Mb. Menière	CI422 / CP810	26	29	20	-	100	90
12	f	62	postCIU	unknown	CI422 / CP810	50	62	8	+	50	35
13	f	71	postCIU	ISSHL	Concerto / Opus 2	61	69	23	+	100	45
14	f	68	postCIU	ISSHL	Concerto / Opus 2	65	68	7	-	100	70
15	f	67	postCIU	cholesteatoma	CI422 / CP810	-	65	20	-	90	20
16	f	69	postCIU	noise-induced	Concerto / Opus 2	46	67	14	-	100	70
17	f	72	postCIU	unknown	HiRes90K / Harmony	-	70	20	+	100	60
18	f	69	postCIU	otitis media	Concerto / Opus 2	-	68	9	+	100	50
19	f	63	postCIU	unknown, ear operation	CI422 / CP810	56	62	10	+	100	75
20	f	51	postCIU	acoustic neuroma	CI422 / CP810	30	50	21	+	100	55
21	f	65	postCIU	Mb. Menière	Concerto / Opus 2	63	63	21	-	100	35
22	m	64	postCIU	otitis media, fami-lial	Concerto / Opus 2	58	63	14	+	100	80
23	m	79	postCIU	poss. hereditary, zoster oticus	CI422 / CP810	77	78	13	-	100	30

	24	m	79	postCIU	blood flow disorder, ISSHL	CI422 / CP810	77	78	11	-	100	85
	25	m	72	postCIU	operation due to cholesteatoma	Concerto / Opus 2	55	71	11	+	100	45
	26	m	74	postCIU	ear operation	Concerto / Opus 2	-	72	14	-	100	50
	27	m	69	postCIU	ISSHL, cholesteatoma	Concerto / Opus 2	67	68	11	+	90	25
	28	m	72	postCIU	ISSHL	CI422 / CP810	69	71	9	-	100	60
	29	m	61	postCIU	cholesteatoma	CI422 / CP810	58	60	12	+	100	80
	30	m	76	postCIU	ISSHL	HiRes90K / Harmony	52	75	16	+	100	55
	31	m	71	postCIU	unknown	CI422 / CP810	66	70	20	-	100	60
	32	m	55	postCIU	unknown	Concerto / Opus 2	53	54	7	+	100	20
	33	m	46	postCIU	unknown	Concerto / Opus 2	36	46	5	-	90	10
	34	m	68	postCIU	ISSHL	CI512 / CP810	66	66	25	+	100	80
	35	m	71	postCIU	Mb. Menière	CI512 / CP810	67	68	30	-	100	90
	36	m	52	postCIU	ISSHL	Concerto / Opus 2	45	50	14	+	100	50
	37	m	51	postCIU	progressive sensorineural HL	CI512 / CP810	47	50	20	+	100	90
	38	m	65	postCIU	noise-induced	CI512 / CP810	63	63	23	-	90	20
x			**65**				**56,6**	**64,1**	**14,3**		**93,2**	**51,7**
	39	f	31	preCIU	unknown, ISSHL	left: CI34RE / CP810 right: CI512 / CP810	3	25	78	+	100	70
	40	f	25	preCIU	hereditary	HiRes90K / Harmony	0	24	13	+	100	35
	41	f	40	preCIU	unknown	left: CI422 / CP810 right: CI512 / CP810	2	37	29	+	50	30
	42	f	25	preCIU	unknown	CI422 / CP810	0	24	8	+	10	5
	43	f	42	preCIU	epidemic parotitis 1.Lebensjahr	Concerto / Opus 2	3	36	78	+	60	0
	44	f	30	preCIU	otitis media	left: M Pulsar/ Opus 2 right: Concerto/ Opus 2	1	22	103	+	70	10
	45	f	54	preCIU	premature birth, complications	CI422 / CP810	0	54	8	+	50	10
	46	m	47	preCIU	unknown, innate	Concerto / Opus 2	0	46	15	+	20	0
	47	m	35	preCIU	unknown, otitis media	CI422 / CP810	3	33	8	-	100	20

	48	m	42	preCIU	meningitis	Concerto / Opus 2	0	41	9	-	10	0
	49	m	23	preCIU	otitis media	CI422 / CP810	2	22	11	+	70	20
	50	m	70	preCIU	meningitis	Concerto / Opus 2	3	69	11	+	70	0
	51	m	23	preCIU	Mb. Gusher	Concerto / Opus 2	3	20	28	+	70	35
	52	m	29	preCIU	unknown, poss. innate	CI422 / CP810	0	28	11	+	100	30
	53	m	28	preCIU	unknown, innate	CI512 / CP810	2	26	22	+	100	70
$\bar{x}$			**36**				**1,5**	**33,8**	**28,8**		**65,3**	**22,3**
